# Increasing strength and conductivity of Cu alloy through abnormal plastic deformation of an intermetallic compound

**DOI:** 10.1038/srep30907

**Published:** 2016-08-04

**Authors:** Seung Zeon Han, Sung Hwan Lim, Sangshik Kim, Jehyun Lee, Masahiro Goto, Hyung Giun Kim, Byungchan Han, Kwang Ho Kim

**Affiliations:** 1 Structural Materials Division, Korea Institute of Materials Science, Changwon 642-831, Korea; 2 Department of Advanced Materials Science and Engineering, Kangwon National University, Chuncheon 200-701, Korea; 3 Department of Materials Engineering and Convergence Technology, ReCAPT, Gyeongsang National University, Chinju 660-701, Korea; 4 Department of Materials Science and Engineering, Changwon National University, Changwon 641-773, Korea; 5 Department of Mechanical Engineering, Oita University, Oita 870-1192, Japan; 6 Gangwon Regional Division, Korea Institute of Industrial Technology, Gangneung 210-340, Korea; 7 Department of Chemical and Biomolecular Engineering, Yonsei University, Seoul, 120-749, Korea; 8 School of Materials Science and Engineering, Pusan National University, Busan 609-735, Korea

## Abstract

The precipitation strengthening of Cu alloys inevitably accompanies lowering of their electric conductivity and ductility. We produced bulk Cu alloys arrayed with nanofibers of stiff intermetallic compound through a precipitation mechanism using conventional casting and heat treatment processes. We then successfully elongated these arrays of nanofibers in the bulk Cu alloys to 400% of original length without breakage at room temperature using conventional rolling process. By inducing such an one-directional array of nanofibers of intermetallic compound from the uniform distribution of fine precipitates in the bulk Cu alloys, the trade-off between strength and conductivity and between strength and ductility could be significantly reduced. We observed a simultaneous increase in electrical conductivity by 1.3 times and also tensile strength by 1.3 times in this Cu alloy bulk compared to the conventional Cu alloys.

The pursuit of high-performance systems and products to satisfy the human desire for versatile functions has been achieved continuously by upgrading materials to have various useful properties. However, designing such materials has been delayed since it requires overcoming restriction formulated by our daily experience. For instance, improvement of mechanical strength and electric conductivity is a long-standing dream of material scientists because they were accepted as mutually exclusive properties.

Metal alloys with high versatility have been developed to satisfy reliability, formability, and power or signal transportation requirements through the introduction of new concepts and microstructures^1^,^2^,^3^,^4^,^5^,^6^,^7^. Recently, new design principles and concepts were emerging to improve ductility without degrading the mechanical strengths using ultra-fine-grained structures^1^,^2^. Even it was attempted to enhance electrical conductivity with the mechanical strength and ductility by artificially introducing nano-scale twins in pure Cu^4^,^5^,^6^. Furthermore, it was reported that bimodal intermetallic compounds in steels^7^ and marble structures in Ti alloys^3^ yielded increase both strength and ductility at the same time.

In this study, we demonstrated new approach to enhancing mechanical strength and electrical conductivity, which is even applicable for conventional metal making technique of thermo-mechanical manufacturing alloys, such as precipitation hardening^8^,^9^,^10^.

According to the conventional theory of particle strengthening, the mechanical properties of metal alloys depend on particle size and interparticle distance in the matrix^11^; i.e., for a given volume fraction of precipitates in the matrix, smaller particles and shorter distances tend to yield higher strength. Thus, the key question is how to induce the formation of smaller precipitates uniformly distributed over the matrix. For example, Cu–Ni–Si alloy made by continuous precipitation (CP) (generally known as normal precipitation) hardening method is the good example showing relatively high strength and electrical conductivity^12^,^13^,^14^.

We focused that discontinuous precipitation (DP) often occurs during the precipitation-hardening process mostly unexpectedly during overaging process, which was known to substantially decrease the mechanical strength of the parent materials^15^,^16^,^17^,^18^,^19^,^20^,^21^. Typically the DP process increases precipitate particle sizes and interparticle distances within the material through the grain-boundary migration and the transformation of precipitate morphologies into lamellar structures^16^,^20^. The CP is therefore generally preferred over the DP to increase the strength of precipitation-hardened alloy in the aging processes based on the morphological point of view in particle strengthening theory^16^,^17^,^18^,^19^,^20^,^21^. Even though the DP is considered to detrimental to the mechanical property, it can be beneficial in improving the electrical conductivity of metal by lowering the concentration of solute in the matrix^21^. The idea is to utilize the DP as a nano-sized strengthening fiber such as in a composite material since it has a form of long fiber and is composed of stiff intermetallic compound. Unlike the conventional composite material, the nano-scale fibers of precipitate are formed in-situ from solid solution by the decomposition process^17^.

We designed an alloy in which nanofibers of the Ni_2_Si intermetallic compound were embedded in the Cu matrix by intentional DP process, as described in the supplementary material Figs S1–S3. We identified that small amount of Ti can substantially crank the driving force for DP up leading to kinetically fast formation of precipitations in Cu–Ni–Si alloy without changing the structure and composition of Ni_2_Si intermetallic compound fibers in Cu matrix (Supplementary Fabrication of Materials, see also Supplementary Fig. 3). Therefore, Ti simply acted as a seed element to accelerate the formation of DP without changing the microstructural morphology in Cu-Ni-Si alloy.

To make the alloys with the microstructure of either fully CP or DP, two Cu-6Ni-1.5Si alloys, without and with 0.1% Ti, were solution heat treated, cooled with different cooling rates and subsequently aged for 7 hours. The Cu–6Ni–1.5Si alloy was solution heat treated and slowly cooled in an open air for the microstructure of CP with the precipitates uniformly distributed in the matrix (Fig. 1(a,b)). For the formation of DP with non-uniform distribution of precipitates through the matrix, on the other hand, the Cu–6Ni–1.4Si–0.1Ti alloy was quenched after solution heat treatment (Fig. 1(c,d)). Despite the significant difference in morphology^8^, the composition and crystallographic structures are the same for the CP and the DP in Cu matrix (Fig. 1 and Supplementary Fig. S3).

The maximum length and aspect ratio of the Ni_2_Si discontinuous precipitates in the Cu–6Ni–1.4Si–0.1Ti alloy were 10 μm and more than 250, respectively (Fig. 1). Using transmission electron microscopy (TEM) and electron diffraction patterns collected in the longitudinal and transverse directions we confirmed the fibers are the Ni_2_Si discontinuous precipitates of orthorhombic structures (space group *Pbnm* with *a* = 0.706 nm, *b* = 0.499 nm, and *c* = 0.372 nm)^17^. The high-resolution TEM images (Fig. 1g) and digital diffractogram (inset of Fig. 1g) suggested that the precipitates and Cu matrix have fully coherent interfaces with the low values of the lattice mismatch δ^8^ (0.0048 for the minimum δ, with 0.207 and 0.208 nm of the (021) plane of Ni_2_Si and the (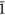
11) Cu plane spacings, respectively).

Given the full distribution of strong fibrous precipitates and the stable interface with a strong coherence, the hardness level (Supple. Matter Fig. S2b) of the alloy with the structure of fully DP was rather disappointing. However, such a strong coherence between the fibrous precipitates and the Cu matrix, the different interface compare to that of general fiber reinforced metal matrix composite might facilitate the alignment of the Ni_2_Si fibers through mechanical deformation and allow the formation of an ideal fiber-arrayed composite. Consequently, the Cu matrix could be reinforced by aligning of the Ni_2_Si nanofibers beyond the upper limit that a fiber-reinforced composite with the morphology of an ideal iso-strain mode could have.

To align fibrous Ni_2_Si intermetallic compound in copper matrix, fully DPed sample was drawn at room temperature with fully CPed counterpart for comparison. We observed no notable morphological change in the Cu–6Ni–1.5Si sample after cold drawing, except that the Ni_2_Si precipitates were merely aligned along the drawing direction (Fig. 2a). However, notable morphology changes in the DPs in the Cu matrix were observed, indicating that the DPs were not only aligned in one direction but also abnormal plastic deformation occurred even at room temperature. Therefore, using cold drawing to 95% reduction of the cross-sectional area, we achieved this alignment and discovered that the diameter and length of brittle Ni_2_Si intermetallic compound decreased to 50% and increased to 400% of their initial values, respectively (Fig. 2(b–d)). Despite the high degree of cold drawing at room temperature (95% drawing, true strain, *η*_Cu alloy_ = 3.0) reducing the diameter of the fibrous precipitates from 13.6 to 6.7 nm (with a drawing ratio of 76%, *η*_precipitate_ = 1.4) and decreasing the average spacing between the precipitates from 87.3 to 26.3 nm (see Fig. 3(e)) we could not identify noticeable defects or cracks in the Ni_2_Si nanofibers. The total true strain accumulated in the specimens after final drawing was just 3 (95% area reduction after drawing), and each pass of drawing was carried out so that the true strain was in the range of 0.04~0.1 (4~10% area reduction, which was used in the conventional drawing sequences). It was therefore believed that dynamic recrystallization did not occur in the present alloys, unlike the alloys with severe plastic deformation (where the total accumulated true strain is beyond 7, and the true strain during one pass is above 1) such as ECAP, ARB and HPT. Only the grain elongation, rather than grain refinement, was observed, as shown in Fig. 2. It was therefore suggested that the strengthening caused by grain refinement due to drawing would be less significant than the other strengthening mechanisms, including work hardening and particle strengthening. This is surprising outcome since the intermetallic nanofibers were elongated by 400% at room temperature by drawing (Supplementary Fig. S4) in spite of its brittle nature (the hardness of a bulky δ-Ni_2_Si intermetallic compound is 620 Hv (6.08 GPa)).

Underlying mechanism enabling the abnormal plastic deformation of such a hard intermetallic compound while aligning them unidirectionally is not well understood. We hypothesized that the phenomenon was related to the stable and coherent interface between the Ni_2_Si nanofibers and the Cu matrix; thus, the plastic deformation of the stiff intermetallic compounds could track the movement of the Cu matrix during the cold drawing process.

To understand the effect of the Ni_2_Si nanofiber precipitates on the mechanical strength, we compared the tensile behavior of the Cu–6Ni–1.5Si alloy with CPs and that of Cu–6Ni–1.4Si–0.1Ti alloy with DPs of Ni_2_Si. Before the drawing process, the tensile strength and the electrical conductivity were measured to be 630 MPa and 46% IACS, and 570 MPa and 50% IACS for the Cu alloys with CPs and DPs, respectively (Fig. 3(a)). After 95% cold drawing, the tensile strength, electrical conductivity and ductility of the Cu–6Ni–1.4Si–0.1Ti alloys were measured as 1,050 MPa, 43% IACS, and 4.7%, whereas 820 MPa, 37% IACS, and 3.6% for the Cu–6Ni–1.5Si. The strength of Ni_2_Si–Cu alloy with embedded nanofibers increased significantly while maintaining its high conductivity and ductility. The increase in the tensile strength in the DP alloy is impressive even though it has lower values than that of the CP alloy before the drawing.

Based on the assumption that the strain hardening caused by the drawing process should be similar for both alloys, we propose that the tensile strength of the Cu–6Ni–1.4Si–0.1Ti alloy was originated from the plastic deformation of the stiff Ni_2_Si nanofiber arrays during cold drawing, which were coherently interfaced with the Cu matrix (Fig. 1g and Supplementary Fig. S5). Specifically the extraordinary high strength in the DP alloy after drawing was likely caused by the decreased radii and interdistances of the layered nanofibers via the abnormal plastic deformation of the stiff Ni_2_Si intermetallic compound as shown Fig. 4. This plastic deformation of fibers might have occurred during the tensile test. The work hardening rate of DP was lower than that of CP during tensile test, suggesting that the increase in dislocation density during plastic deformation of the alloy was suppressed by the plastic deformation of the DPed Ni_2_Si intermetallic compound fibers in the metal matrix. Therefore, the strain of the alloy was absorbed or evenly distributed by the abnormal plastic deformation of the intermetallic compound fibers. Although the deformation was smaller than during the drawing step, it was likely a key factor in increasing the ductility by decreasing the density of the dislocations in the DP and drawn alloy as shown Fig. 5. The results in this study indicate that an unprecedented combination of high tensile strength, ductility, and conductivity has been achieved along the drawing direction. The tensile strengths and conductivities obtained in this work were significantly improved over previously reported values^14^,^16^,^21^,^22^,^23^,^24^,^25^,^26^,^27^,^28^,^29^,^30^ (see Fig. 3b).

In conclusion, we found the extraordinarily strong Cu alloy with surprisingly high electric conductivity. Underlying mechanisms were based on the abnormal plastic deformation of DPs, Ni_2_Si nanofibers in the Cu matrix over cold drawing at room temperature, and dilution of solute atoms in the Cu matrix by fast dissolution during the DP process. This work may also provide new strengthening concept for a wide class of alloys that can undergo precipitation hardening; such alloys would otherwise suffer from degradation in mechanical strength because of the formation of cellular-type discontinuous precipitates during aging.

## Methods

Samples from Cu–6Ni–1.5Si and Cu–6Ni–1.4Si–0.1Ti alloy ingots that were 20- or 40-mm thick were fabricated by vacuum induction melting. To induce DP, the ingots were hot-rolled at 980 °C to a 6 or 10 mm thick plate, solution heat-treated at 980 °C for 2 h, and subsequently aged at 500 °C for 1/6, 1/2, 1, 3, 6, or 7 h. To induce normal precipitation, the Cu–6Ni–1.5Si alloy was cooled in air, whereas the Cu–6Ni–1.4Si–0.1Ti alloy was water quenched after the solution treatment and before aging. A drawing process was used to align the fiber-like precipitates in the overaged Cu–Ni–Si samples.

Cylindrically machined samples with diameters of 5 or 6.5 mm were drawn at room temperature to a 95% reduction in their cross-sectional area (true strain, η = 3.0). Their electrical conductivity was measured using an electrical conductivity meter for the rolled samples and a resistivity meter for the drawn samples. The microhardness was measured using a Vickers hardness tester under a 200-g load. Tensile tests were conducted with plate and wire samples, and the measurements were conducted using a 12.5-mm gauge at a nominal strain rate of 1.3 × 10^−3^/s with a universal testing machine.

The grain morphology and coarse secondary phase particles of the Cu–Ni–Si samples were observed using an optical microscope and a scanning electron microscope. A 200-kV field-emission TEM equipped with an EDS detector was used to examine the precipitates and the secondary-phase particles. More information is available in the Experimental section in the Supplementary Information regarding the experimental conditions and procedures, electrical conductivity, mechanical tests, and microstructural analysis.

## Additional Information

**How to cite this article**: Han, S. Z. *et al*. Increasing strength and conductivity of Cu alloy through abnormal plastic deformation of an intermetallic compound. *Sci. Rep.* **6**, 30907; doi: 10.1038/srep30907 (2016).

## Supplementary Material

Supplementary Information

## Figures and Tables

**Figure 1:**
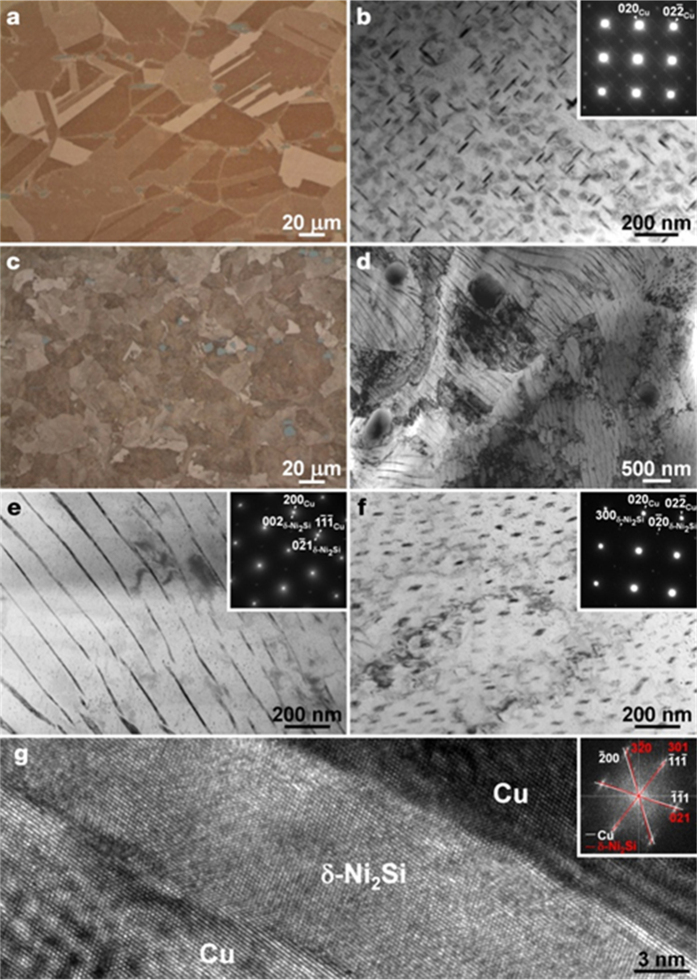
(**a**) A sample of Cu–6Ni–1.5Si alloy that was air-cooled after solution heating and aging at 500 °C for 7 h. The image shows a typical microstructure of a normally precipitated alloy. (**b**) Bright-Field (BF) TEM micrograph showing that the Cu–6Ni–1.5Si alloy has uniformly distributed disc-type Ni_2_Si precipitates. (**c**) A sample of Cu–6Ni–1.4Si–0.1Ti alloy that was water-quenched after being solution treated and then heat treated as in Fig. 2a, but showing a different microstructure, with grains that appear to be tarnished. (**d**) BF TEM micrograph showing lamellar and elongated Ni_2_Si particles. The difference in the driving force for the precipitation in the alloy is shown to produce significant morphological alterations and a considerable change in mechanical strength. BF TEM images and selected area diffraction patterns in (**e**) longitudinal and (**f**) transverse directions confirm the fiber-like Ni_2_Si precipitates. (**g**) High-resolution TEM image with a digital diffractogram, showing the precipitates exhibiting a δ-Ni_2_Si structure and Ni_2_Si in the Cu matrix exhibiting the orientation relationships of {111}_Cu_//{301}_Ni2Si_, {111}_Cu_//{021}_Ni2Si_, and {200}_Cu_//{320}_Ni2Si_.

**Figure 2:**
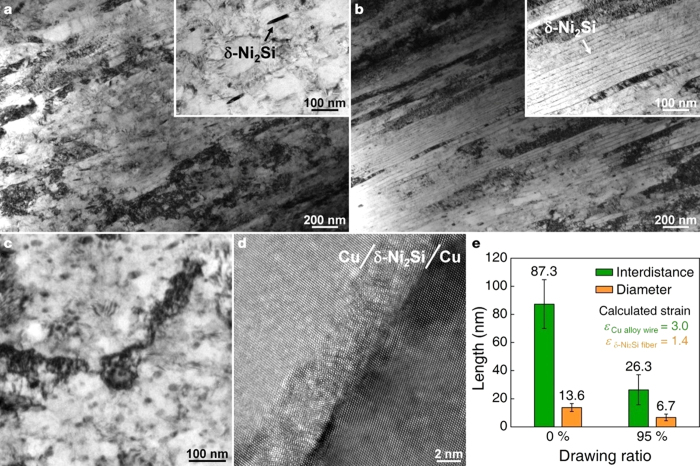
The TEM microstructures of the cold-drawn alloys at room temperature show the uniformly arrayed precipitates and (**a**) typical plastically deformed microstructure in Fig. 3a. The Ni_2_Si precipitates were not deformed during drawing (inset in Fig. 3a). However, with the same drawing conditions as the normally precipitated alloy, (**a**) fully discontinuous precipitated alloy showed a significantly different morphology (**b**). The Ni_2_Si precipitates were aligned and were plastically deformed along the drawing direction (**b**) and the transverse direction (**c**). The average distance between the precipitates and their diameters decreased during drawing. Even under a drawing strain (*η*) of approximately 3.0 (i.e., a 95% drawing ratio), the hard and brittle Ni_2_Si intermetallic compounds plastically deformed without breaking (Fig. 3(b–d)). The change in the interparticle distance and the diameter of the particles in the Cu–Ni–Si–Ti alloys is shown in (**e**). The drawing ratio of the precipitates was approximately 76% (*η*_precipitate_ = 1.4), which indicates that the Ni_2_Si intermetallic compounds were elongated by up to four times their initial length during the conventional cold-drawing process.

**Figure 3:**
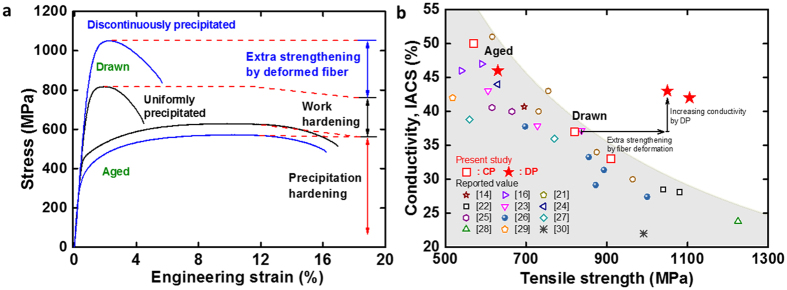
(**a**) After aging, the tensile strength of the uniformly precipitated alloy had a higher strength than the discontinuously (cellular) precipitated alloys, because of smaller-sized and uniformly distributed second-phase particles in the normally precipitated alloy. However, after drawing, the tensile strength of the discontinuously precipitated alloy was higher than that of the uniformly distributed precipitated alloy. As expected, the change in conductivity in the continuously and discontinuously precipitated alloy showed just 9% and 7% IACS after drawing with 95% area reduction respectively, which means that strain hardening occurred in both alloys to a similar degree. (**b**) Interestingly, however, the increase in tensile strength of the discontinuous cellular precipitated alloys represented a higher value of the strength, beyond the expectation from strain hardening. The additional strengthening from the aligning and abnormal plastic deformation of the intermetallic compounds during drawing reached a value of 290 MPa with a higher conductivity, which was a unique property of overaged discontinuously precipitated alloys. On further drawing to 97.5%, the tensile strength and conductivity of the DP samples reached 1105 MPa and 41% IACS, respectively.

**Figure 4:**
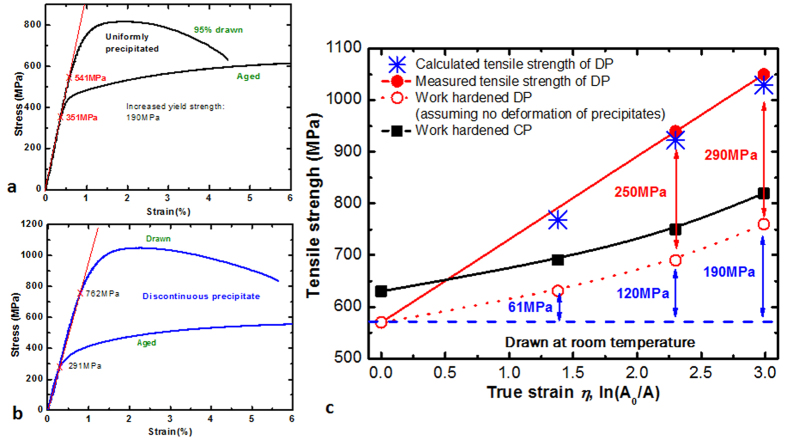
The increased yield strengths of normally and discontinuously precipitated alloy after being drawn to 95% of their original cross-sectional areas were 190 (**a**) and 471 MPa (**b**), respectively. The shape of the normal precipitates in the matrix could not be changed during drawing; the strengthening of the normally precipitated alloy was concluded to primarily occur due to work hardening during the drawing process. Assuming that both the normally and discontinuously precipitated alloys experienced the same work hardening during the drawing process, the total increase in the strength in the discontinuously precipitated alloy (solid red circles in **c**) was significantly higher than the expected value (open red circles in **c**) after drawing. Additionally, the calculated increase in the strength due to the decrease in the radius and the interparticle distance between the fibers during drawing showed good agreement with the measured value. The additional strengthening of the discontinuously precipitated alloy is therefore concluded to have originated from the plastic deformation of the Ni_2_Si intermetallic fibers during drawing.

**Figure 5:**
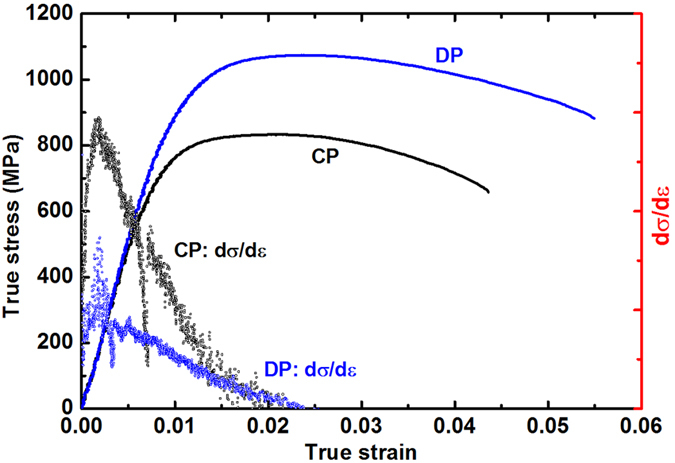
The rate of work hardening of the fully DP structure showed a lower value than that of the other alloys. This result indicates that the work hardening of the Cu matrix was interrupted by the plastic deformation of the precipitated fibers; the dislocations generated during the tensile test were therefore absorbed by the plastic deformation of the intermetallic compound fibers.
