# Supplementary material for: Increasing strength and conductivity of Cu alloy through abnormal plastic deformation of an intermetallic compound
Source: Sci Rep. 2016 Aug 4;6:30907. doi: 10.1038/srep30907 (PMC4973219; doi:10.1038/srep30907)
Supplement: Supplementary Information [file srep30907-s1.pdf]

Supplementary materials for

Increasing strength and conductivity of Cu alloy through abnormal plastic deformation of an intermetallic compound

Seung Zeon Han[1], Sung Hwan Lim[2]*, Sangshik Kim[3], Jehyun Lee[4]*,
Masahiro Goto[5], Hyung Giun Kim[6], Byungchan Han[7], and Kwang Ho Kim[8]

[1]Structural Materials Division, Korea Institute of Materials Science, Changwon 642-831, Korea
[2]Department of Advanced Materials Science and Engineering, Kangwon National University, Chuncheon 200-701, Korea
[3]Department of Materials Engineering and Convergence Technology, ReCAPT, Gyeongsang National University, Chinju 660-701, Korea
[4]Department of Materials Science and Engineering, Changwon National University, Changwon 641-773, Korea
[5]Department of Mechanical Engineering, Oita University, Oita 870-1192, Japan
[6]Gangwon Regional Division, Korea Institute of Industrial Technology, Gangneung 210-340, Korea.
[7]Department of Chemical and Biomolecular Engineering, Yonsei University, Seoul, 120-749, Korea
[8]School of Materials Science and Engineering, Pusan National University, Busan 609-735, Korea
*To whom correspondence should be addressed.
E-mail: shlim@kangwon.ac.kr, and ljh@changwon.ac.kr

Materials and Methods

A. Fabrication of materials

Pure Cu and Si with purities of 99.99% and Ni with a purity of 99.9% were used to prepare the materials investigated in this study. The nominal compositions of the alloys were designed to increase the precipitation driving force via Ti addition, as described in Supplementary Fig. 1. The composition of the two alloys is shown in Supplementary Table 1. Cu–Ni–Si alloy ingots with and without additional Ti were fabricated from 40- and 20-mm-thick cast ingots, respectively, via vacuum induction melting. The Cu–6Ni–1.5Si ingot without added Ti could not be hot-rolled because of severe hot cracking. It was consequently only cold-rolled to a 6-mm-thick plate. By contrast, the Cu–6Ni–1.4Si–0.1Ti ingot could be hot-rolled at 980°C to a 6- or 20-mm-thick plate. To eliminate the thermomechanical history of the specimens, both the cold- and hot-rolled plates were subsequently solution heat-treated at 980°C for 2 h, followed by aging at 500°C for 1/6, 1/2, 1, 3, or 6 h. Finally, the two types of specimens, in which the precipitates were uniformly distributed(CP, continuous precipitation) and fully precipitated discontinuously(DP, discontinuous precipitation), were air cooled and water quenched after solution treatment at 980°C for 2 h. To align the fiber-like precipitates in a single direction, a drawing process was applied to both alloys that were aged under the same conditions of 500°C for 7 h after being solutionized. Cylindrically machined specimens with diameters of 5 and 6.5 mm, were drawn at room temperature with a 95% reduction in their cross-sectional area (true strain, η = 3.0).

To create monolithic δ-Ni_2Si intermetallic compounds, the composition of Ni/Si was weighed to an atomic ratio of 2 with a total weight of 40 g; the material was then fabricated by vacuum arc melting and homogenized at 950°C for 1 h.

B. Property evaluation and microstructural analysis

The electrical conductivity of the plate specimens was measured with an electrical conductivity meter (Sigmascope SMP-10, Fischer, Germany) and that of the wire specimens was measured with a resistivity meter (Portable double bridge 2769, Yokogawa, Japan). The microhardness of the specimens was measured with a Vickers hardness tester (MXT70, Matsuzawa, Japan) under a 200-g load. Tensile tests were performed with a gage length of 12.5 mm at a nominal strain rate of 1.3×10^{-3}/s on a universal testing machine (EZ-L, Shimadzu, Japan). The grain morphologies and coarse secondary-phase particles of the as-cast, solutionized, and aged specimens were observed with an optical microscope (GX51, Olympus, Japan) and a scanning electron microscope (JSM-5800, JEOL, Japan). The precipitates and secondary phase particles were investigated using a transmission electron microscope (JEM-2010, JEOL, Japan), a 200-kV field-emission transmission electron microscope (JEOL-2100F, JEOL, Japan) equipped with an energy-dispersive X-ray spectroscopy (EDS) detector, and a scanning transmission electron microscope. The TEM specimens were prepared to have a 3-mm diameter in the form of 100-μm-thick disk-type plates via mechanical polishing with a digitally enhanced precision specimen grinder (DEPS-101, Total Solution). These samples were then jet-polished with a Tenupol-5 (Struers) in an etchant solution composed of 250 mL of phosphoric acid, 500 mL of distilled water, 250 mL of ethanol, 50 mL of propanol, and 5 g of urea (D2). Subsequently, before being inserted into the TEM chamber, the specimens were trimmed and cleaned by precision ion polishing with a 691 PIPS device (Gatan) and subjected to plasma cleaning with a Fischione 1020 cleaner (Fischione).

C. Preparation of fully DP specimen

The driving force for precipitation needs to be increased and lead fast formation of precipitation to make DP in Cu–Ni–Si alloy. Ti was chosen for these purposes, as shown in Supplementary Fig. S1. The Cu–6Ni–1.5Si alloys were prepared with and without the substitution of 0.1 wt% Ti to Si. The two alloys were aged at 500 °C after being solutionized at 980 °C for 1 hr. Ti was observed to considerably affect the mechanical and electrical behaviors of the Cu–Ni–Si alloys (Fig. S2). The electrical conductivity improved by 60% in terms of the International Annealed Copper Standard (IACS), and the mechanical hardness decreased by 40% upon the substitution of 0.1 wt% Si with Ti in Cu–6Ni–1.5Si (Fig. S2(a and b)). The increase in electrical conductivity can be explained by the improved precipitation kinetics of Ni_2Si, which rapidly removes the solutes that resist electron movement in Cu matrix. The decrease in hardness was due to the accelerated thermal aging of discontinuous cellular-type precipitates. As evident from Fig. S2(c and d), small amounts of Ti solute considerably increased the density of the blackish grains with a cellular structure compared with the density of the grains in Cu–6Ni–1.5Si without Ti. Consequently, we observed that the addition of Ti accelerated the precipitation rate of the intermetallic compound Ni_2Si, leading to an improvement in electrical conductivity; however, a significant degradation in the mechanical strength of the Cu–Ni–Si alloys has also occurred because of excessive formation of discontinuous precipitates.

As described figure S1, it was hypothesized that the Ti has a tendency to make the SiTi intermetallic compound during aging, and these stable compounds might act as inoculants of growing discontinuous precipitation. Therefore, the grown Ni_2Si intermetallic compounds did not show any compositional or crystallographical changes compare to Cu-6Ni-1.5Si alloy as shown figure S3.

The bulky monolithic intermetallic compounds which has the same composition with CPed or DPed phase in Cu-Ni-Si alloy matrix was made by vacuum arc melting and successive homogenization, and it was proved to δ-Ni_2Si as shown figure S4 and its hardness was 620 Hv (6.08 GPa).

D. Calculating unexpected strengthening of DPed alloy after drawn

To describe the mechanism of the unexpectedly high strengths in the discontinuously precipitated and drawn alloy, we applied the particle strengthening principle (*15, 16*). In this theory, the mechanical strength due to dispersed particles in a matrix is described by Eq. (1):

$$\boldsymbol{\tau = Gb / (L - 2r) = Gb/\lambda} \qquad (1)$$

where τ, G, b, L, λ and r are the shear stress, the shear modulus, the Burgers vector, the interparticle distance, the effective particle space and the particle radius, respectively. Parameter λ in Eq. (1) may be applied to a spherical particle, and Eq. (1) can be converted into another form of Eq. (2) when long fibers are arranged in the matrix, as shown in Figs. 2e and 2f and Supplementary Fig. S5:

$$\mathbf{L{-}2r = \lambda} = \left(\frac{\mathbf{1.905}}{\sqrt{f}} - \mathbf{2}\right)\boldsymbol{r} \qquad (2)$$

In Eq. (2), the effective fiber spacing was assumed to be linearly dependent on the radius of the fiber; thus, r was substituted for λ in Eq. (1). For longitudinally arrayed fibers along the tensile loading direction, and when slipping occurs at 45° relative to the normal plane, λ should be √2 times greater. Inserting λ into Eq. (1) and using the shear modulus (4.5×10^4 MPa) and

Burgers vector (0.256 nm) of pure Cu metal (*36*); a theoretical volume fraction 0.085; and a measured radius of r_0 = 6.8 nm for Ni_2Si-nanofiber discontinuous precipitates in the Cu–6Ni–1.4Si–0.1Ti alloy, we calculated the shear strength to be 264 MPa. This value can be directly converted to a minimum yield strength of 528 MPa with a maximum Schmid factor of 0.5 in a tensile test.

These results, however, are far from the measured yield strength of 291 MPa, as shown in Fig. 4a and Supplementary Fig. S5b. These differences are attributed to the fact that the morphology of experimental specimens may be significantly different from ideal arrays of nanofibers, as shown in Supplementary Fig. S5. It is impossible to consider real morphology of specimen to strength in Eq. 1 exactly, however, under assumption that composition, volume fraction and shape of detrimental 2nd phase are maintained during drawing, a correction factor k that considers diminution from ideal strength can be introduced into Eq. (1). And Eq.1 with Eq. 2 create Eq. (3):.

$$\boldsymbol{\tau_{aged} = k\ Gb/(\sqrt{2}(1.905f^{-1/2}\text{–}2)r_0)} \quad (3)$$

We observed that the value of τ_{aged} calculated using Eq. (3) for Ni_2Si-nanofiber DP arrays was 131 MPa. The increased shear stress was directly evaluated from the measured yield strength of 291 MPa (Fig. 4a and Supplementary Fig. S6b) minus the yield strength of pure Cu (30 MPa) (*37*) with r_0 = 6.8 nm. Then, k Gb / ($\sqrt{2}(1.905f^{-1/2}$ - 2) is equal to 891 x 10^{-9} MPa·m. A change in the shear strength of the aged alloy due to drawing (Δτ) was then simply calculated by replacing $1/r_0$ with Δ(1/r) in Eq. (3). Using the calculated shear strength of 135 MPa, we determined the minimal enhancement of the yield strength to be approximately 270 MPa due to the decreased radius of the nanofibers after the drawing process. Additionally, the total strength of the DP and drawn alloy is the summation of the strength by DP hardening; the work

hardening during the drawing, which is the same as that in the CP alloy because both alloys have similar compositions, precipitate volume fractions, and hardnesses, as shown Fig. 4b; and the additional hardening provided by the decreased nanofiber radius after drawing (Supplementary Fig. S6c). The calculated total yield and tensile strengths were determined to be 751 and 1030 MPa, respectively, which agree with the measured values of 762 and 1050 MPa, as shown in Fig. 3a and Supplementary Fig. S6.

Supplementary Table S1. Average composition of copper alloys used in this work.

Alloy	Cu	Ni	Si	Ti
Cu-6Ni-1.5Si	bal.	5.94	1.44	-
Cu-6Ni-1.4Si-0.1Ti	bal.	6.20	1.34	0.11

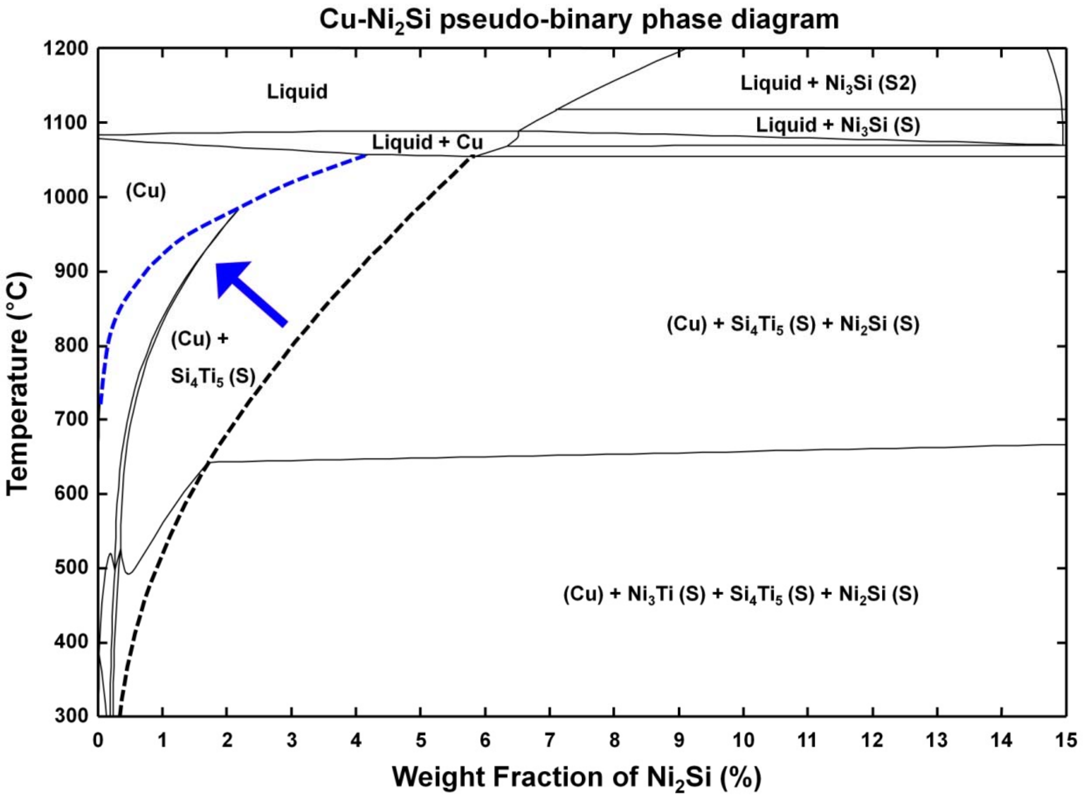

Supplementary Figure S1. The free energy change (Δ*G*) between the solutionized phase and the particle-precipitated matrix is described by the following simple equation:

$$\Delta G = L\,\Delta T / T_m,$$

where *L* is the latent heat energy per molar volume, T_m is the melting point of the alloy and Δ*T* is the temperature difference between the solution line and the aging temperature for a given composition. This diagram shows that, if the solution region decreases, the driving force of the phase separation increases. To describe the thermodynamic effect of the addition of Ti on Ni_2Si precipitation, a pseudo Cu–Ni_2Si phase diagram was calculated using the FactSage software (*38*), where Cu, Ni, Si and Ti binary diagrams were used. The figure shows the calculated Cu–Ni_2Si pseudo-binary phase diagrams with no Ti (black dotted line) and with the addition of 0.1% Ti (blue dotted line). When Ti is added to the Cu–Ni–Si alloy, the single-phase region (Cu) is

shown to considerably decrease. Although the Si_4Ti_5 intermetallic compound is first precipitated upon cooling from the equilibrium phase diagram, in the real solidification process, the Si_4Ti_5 phase is difficult to precipitate exclusively because Ti also has an affinity for Ni (39). The reduced single-phase region at the precipitation temperature indicates that the Cu matrix can be purified to a greater degree, resulting in the removal of more solute elements. In addition, both the supercooling temperature (ΔT) and the supersaturating composition (Δc) increase as the single-phase region decreases in size, meaning that the driving force of the precipitation of the Ni_2Si phase increases.

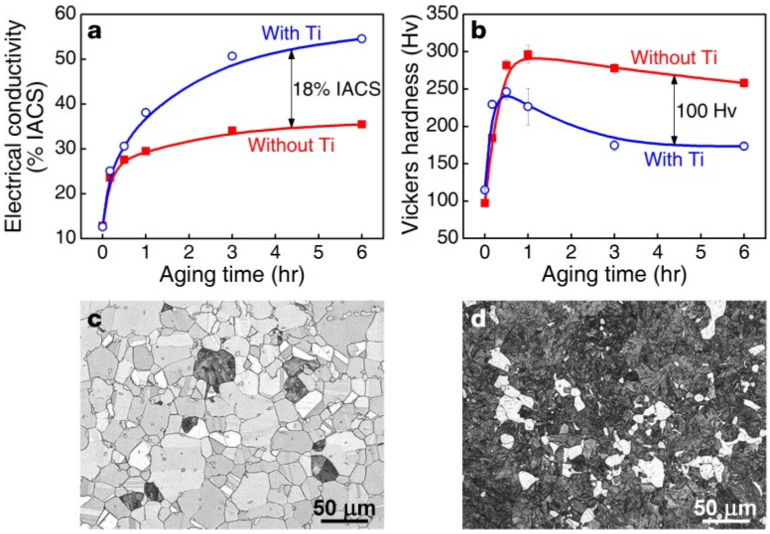

Supplementary Figure S2. The addition of 0.1 wt% Ti has affected the mechanical and electrical behavior of Cu–6Ni–1.5Si samples, and this effect increased with aging: (a) an approximate 60% increase in the electrical conductivity, as represented by the %IACS, and (b) a 40% decrease in hardness were observed after addition of 0.1 wt% Ti to the overaged Cu–6Ni–1.5Si samples. Optical micrographs of (c) Cu–6Ni–1.5Si and (d) Cu–6Ni–1.4Si–0.1Ti samples. After aging at 500 °C for 3 h the samples exhibited significantly different microstructures. The formation of blackish grains shows a cellular structure consisting of extremely high-aspect-ratio precipitates in the matrix; these precipitates are shown in Fig. 1.

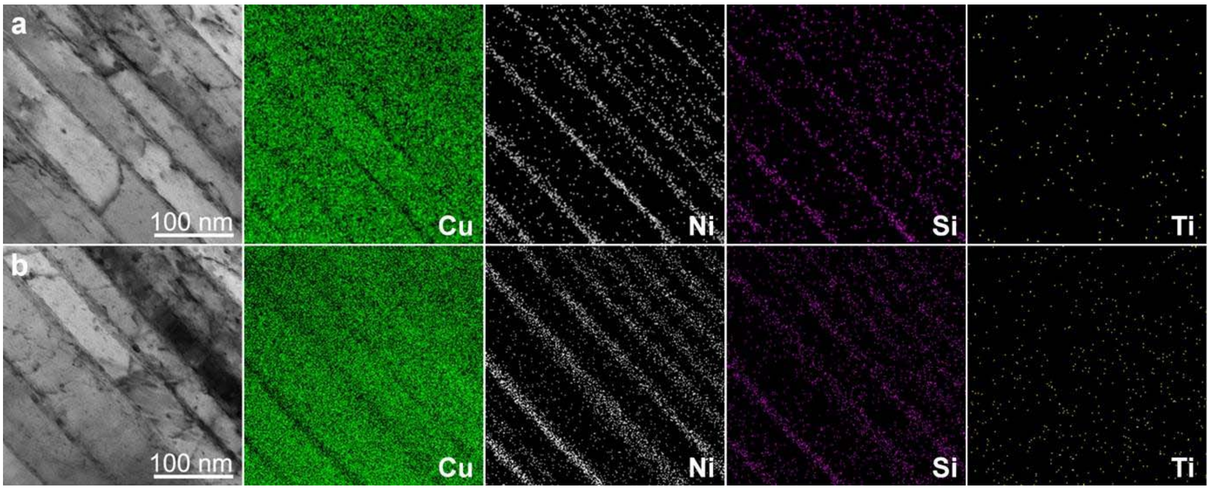

Supplementary Figure S3. EDX results for the Cu–Ni–Si–Ti alloy (a and b), showing that the composition of the discontinuously precipitated δ-Ni_2Si intermetallic compound consists of Ni and Si, without Ti. These results indicate that a small amount of Ti in the Cu–Ni–Si alloy merely accelerate the formation of discontinuous precipitates without changing its composition and structure.

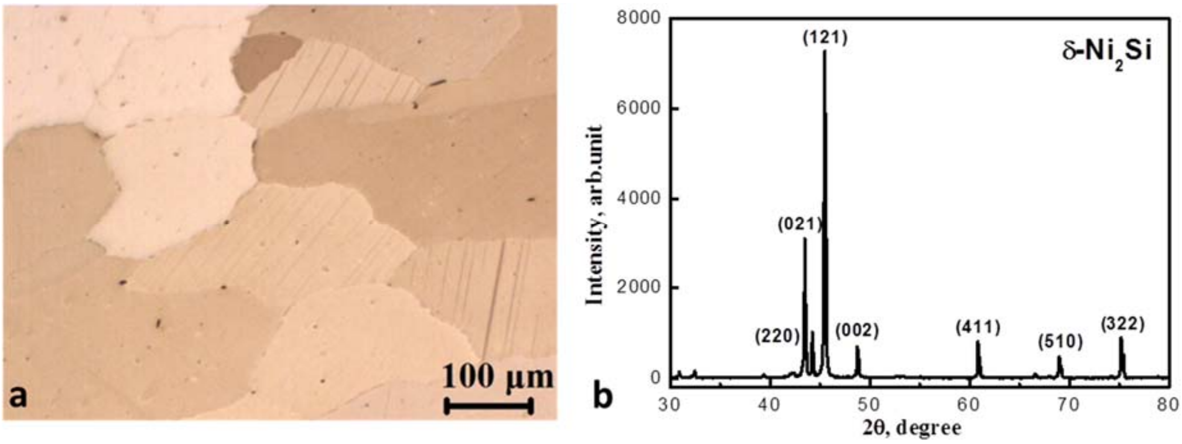

Supplementary Figure S4. Monolithic Ni_2Si intermetallic compound fabricated using vacuum arc melting and homogenization at 950°C for 1 h: (a) optical image of the monolithic δ-Ni_2Si intermetallic compound and (b) evidence for the identification of the material as δ-Ni_2Si on the basis of XRD analysis. The measured Vickers hardness of the δ-Ni_2Si intermetallic compound was 620 Hv (6.08 GPa).

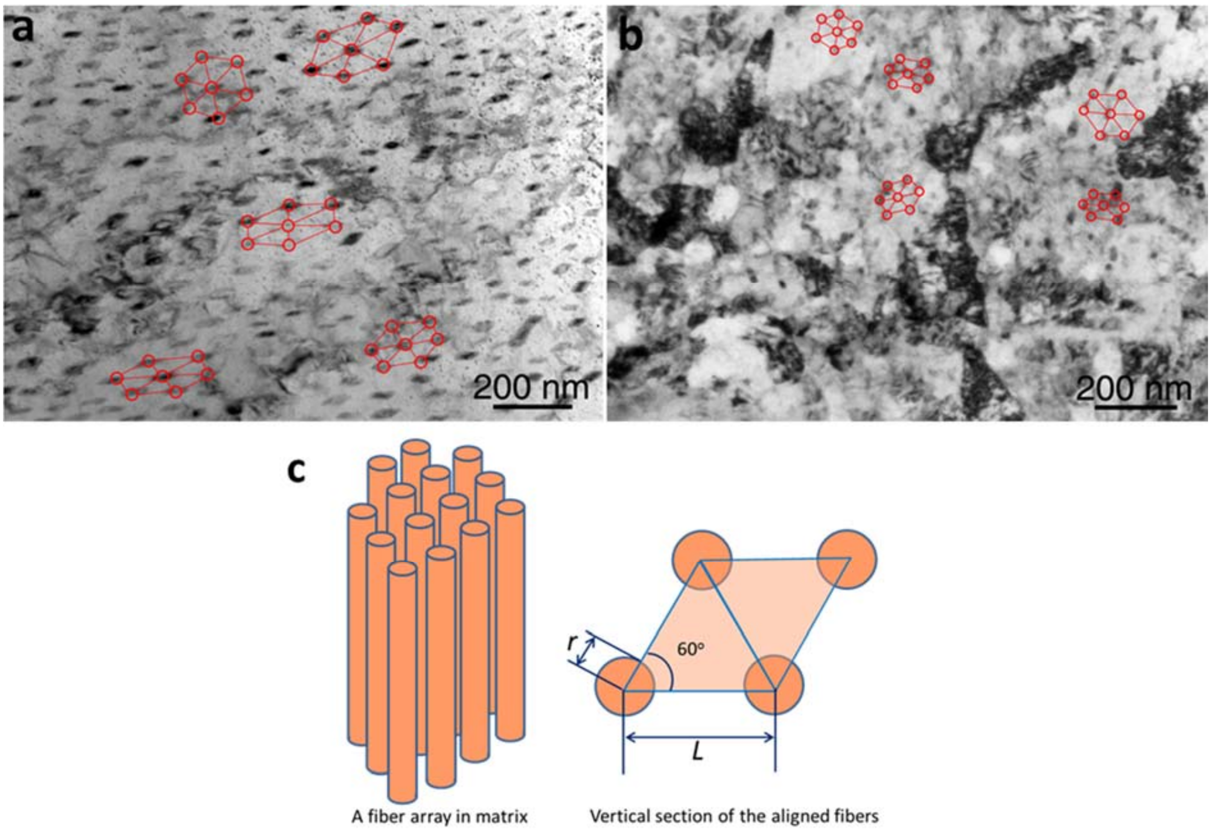

Supplementary Figure S5. According to Figs. 1f and 2c in the manuscript, the δ-Ni_2Si intermetallic compound fibers were arranged closely with other fibers in the aged and drawn alloy (a and b). The rods were assumed to be embedded regularly in the Cu-alloy matrix while a minimum interparticle distance was maintained between the rods, as shown in (c). The relationship between the radius, ***r*** of rod, the interparticle distance, ***L***, and the effective interparticle distance, ***λ***, could be calculated as follows. At a given volume fraction *f* (e.g., the theoretical volume fraction of δ-Ni_2Si is 0.085 in this study) is described by Eq. (s1):

$$f = \pi r^2 / (L^2 \sin 60^o) \quad \textbf{(s1)}$$

and ***L*** and ***λ*** are calculated using Eqs. (s2) and (s3):

$$L = \lambda + 2r = 1.905r / f^{1/2} \quad \textbf{(s2)}$$

$$\lambda = L - 2r = (1.905 / f^{1/2} - 2)r \quad \textbf{(s3)}$$

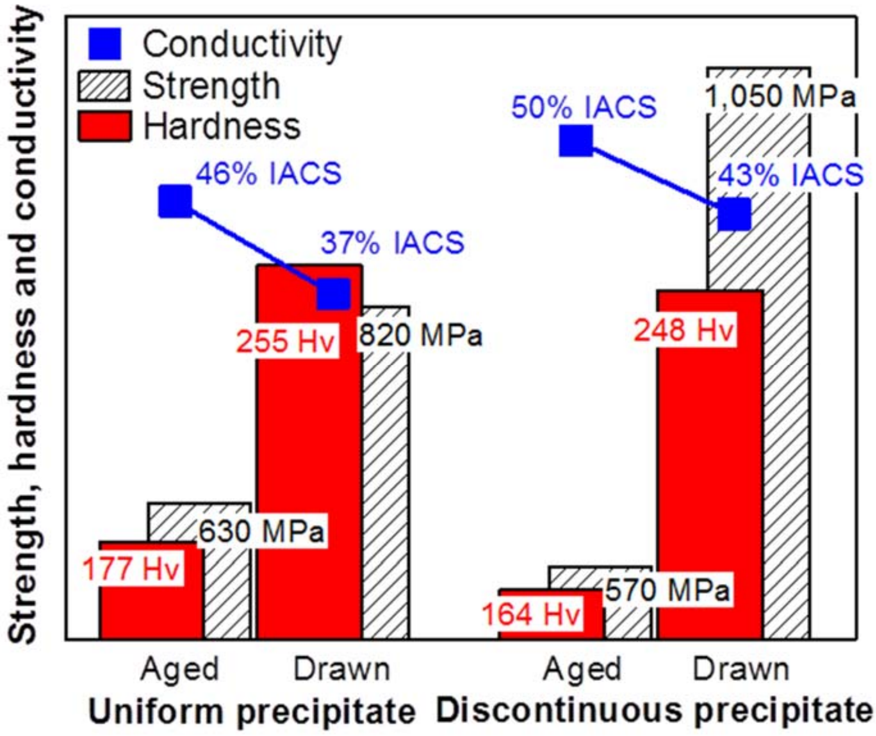

Supplementary Figure S6. The change in conductivity and hardness during drawing showed similar values for each precipitate, meaning that the magnitude of strain hardening was similar for both alloys. However, the increase in tensile strength of discontinuous cellular precipitated alloys after drawing was substantially greater than that of uniform precipitate, which was much more than expected by strain hardening. Such a high tensile strength was believed to be mainly due to the alignment by abnormal plastic deformation and the successive reduction in the radius of precipitate and the inter-distance between them.

References and note for Supplementary Information

1. E. Orowan, Symposium on Internal Stresses in Metals and Alloys (Institute of Metals, 1948).
2. T. H. Courtney, Mechanical Behavior of Materials 2nd edn 232 (Waveland Press, 2005).
3. J. Lee et al., Correlation of the microstructure and mechanical properties of oxidedispersion-strengthened coppers fabricated by internal oxidation, Metall. Mater. Trans. A 35, 493-502 (2004).
4. ASM International, Atlas of Stress-Strain Curves 2nd edn (ASM International, 2002).
5. C. W. Bale et al. FactSage Thermochemical Software and Databases, Calphad 26, 189-228 (2002).
6. S. Z. Han, K. Euh, S. H. Lim, S. Kim, Effect of Ti addition on electrical conductivity and mechanical properties of Cu-Ni-Si alloys, J. of the JRICu 49, 176-181 (2010).
